# Saturated fatty acids accelerate linear motility through mitochondrial ATP production in bull sperm

**DOI:** 10.1002/rmb2.12381

**Published:** 2021-05-06

**Authors:** Md. Mazharul Islam, Takashi Umehara, Natsumi Tsujita, Masayuki Shimada

**Affiliations:** ^1^ Laboratory of Reproductive Endocrinology Graduate School of Biosphere Science Hiroshima University Hiroshima Japan; ^2^ Laboratory of Reproductive Biology Graduate School of Integrated Sciences for Life Hiroshima University Hiroshima Japan; ^3^ Department of Animal Breeding and Genetics Bangabandhu Sheikh Mujibur Rahman Agricultural University Gazipur Bangladesh

**Keywords:** fatty acid transporter, frozen sperm, mitochondria oxygen consumption, mitochondrial β‐oxidation, sperm motility pattern

## Abstract

**Purpose:**

The present study was undertaken to clarify whether bovine sperm could take up fatty acids (FAs) and produce ATP to maintain linear motility.

**Methods:**

Frozen bovine semen was thawed in media containing either lipid mixture (LM) or FAs, and sperm motility was analyzed. The kinetic changes in FA levels in sperm were detected using gas chromatography‐mass spectrometry. The mitochondrial activity of sperm thawed in media containing LM or FAs was analyzed based on the fluorescence intensity of JC‐1 staining and the oxygen consumption rate. FA transporters were observed using whole‐mounted immunofluorescence.

**Results:**

Sperm linear motility was significantly (*P* < .05) increased after thawing in media with LM and FA. Moreover, saturated fatty acids were predominant in sperm thawed in media with LM. Notably, our study revealed that frozen bovine sperm possessed FA transporters in the midpiece where the fluorescence signals were detected after treatment with fluorescence‐tagged FA. Treatment with FA activated electron transport in mitochondria through β‐oxidation.

**Conclusions:**

Sperm linear motility is facilitated by FAs in the thawing media used for frozen bovine sperm. This might provide a new approach for upgrading the artificial insemination technique used in both livestock animals and human infertility care.

## 1 INTRODUCTION

Sperm motility is induced by ATP production^1^ and is indispensable for sperm migration to the oocyte to achieve a successful fertilization.^2^ Motility patterns rely on flagellar motion, and a linear motility pattern is induced by symmetrical flagellar motion.^2^, ^3^ Linear motility is essential for sperm migration from the cervix to the uterus, and then to the oviduct, characterized by low lateral amplitude and high straight‐line velocity.^2^, ^4^ In the oviduct, during the fertilization process, hyperactivation is a prerequisite for sperm capacitation, in which the curvilinear velocity and the lateral amplitude are high.^5^, ^6^, ^7^, ^8^

Two main metabolic pathways are required for ATP‐induced sperm motility: glycolysis, which occurs in the sperm tail, and mitochondrial oxidative phosphorylation (OXPHOS), which is activated in the midpiece of sperm.^9^ Rotenone, an inhibitor of complex I in mitochondria, decreased sperm mitochondrial activity and ATP levels with progressive motility and straight‐line velocity, but not lateral amplitude and total sperm motility in boar,^10^ stallion,^11^ and humans.^12^ Further studies revealed that rotenone suppressed sperm capacitation, acrosome reaction, and decreased the fertilization rate in hamsters^13^ and stallions,^14^ indicating that mitochondrial ATP production is crucial for sperm linear motility and hyperactivation.

Remarkably, in boar sperm, it was reported that a decreased glucose level in extender‐induced linear motility boosted OXPHOS, whereas complete removal of glucose from the medium failed to maintain linear sperm motility and mitochondrial activity.^10^ Glucose is used as a substrate not only in glycolysis, but also in the pentose phosphate pathway to generate NADPH, which affects redox homeostasis and is essential to maintain sperm motility.^15^, ^16^ This suggests that pyruvate, which is derived from glucose, is not the main source of acetyl CoA to activate OXPHOS in sperm. It has been reported that acetyl CoA is produced by mitochondrial β‐oxidation from fatty acids.^17^ Oxygen availability and the composition of metabolic substrates cause variation in the metabolic pathways of cells.^18^

It has been reported that, in human sperm, a large proportion of the metabolic proteome is involved in lipid metabolism, including enzymes that participate in β‐oxidation.^19^ Interestingly, mitochondrial β‐oxidation is an active regulator of sperm motility because the inhibition of β‐oxidation by etomoxir led to a decrease in sperm motility.^19^, ^20^ Moreover, it was reported that palmitic acid of saturated fatty acid class improved sperm progressive linear motion and viability of bull sperm when supplemented to the extender,^21^ suggesting that mitochondrial β‐oxidation, for which exogenous fatty acids are the main source of energy, is essential for sperm progressive motility and survivability.

However, the mechanism by which saturated fatty acids protect sperm progressive motility and viability is still unclear. Therefore, in the present study, we assumed that fatty acids might contribute to ATP production via mitochondrial β‐oxidation to induce the sperm linear motility in vitro. First, we focused on sperm motility with an exogenous lipid mixture (LM) in the thawing media of frozen bovine sperm. Second, we tried to analyze the level of the different fatty acids in frozen bovine sperm treated with lipid mixture using GC‐MS. Finally, we studied sperm motility, mitochondrial activity, and the oxygen consumption rate after treatment with fatty acids.

## 2 MATERIALS AND METHODS

### 2.1 Materials

Routine chemicals were obtained from FUJIFILM Wako Pure Chemical Industries, Ltd. (Osaka, Japan) and Nacalai Tesque (Osaka, Japan). Frozen straws of bovine sperm from Holstein bulls were kindly gifted by the Livestock Improvement Association of Japan, INC (Tokyo, Japan).

### 2.2 Sperm preparation and incubation

Frozen bull semen was thawed in water at 37°C and washed twice via centrifugation (600 × *g*, 4 min) with 6 mL of Bracket‐Oliphant (BO) medium.^22^, ^23^ The sperm pellet was resuspended in BSA‐containing BO medium with 0.1% of lipid mixture (LM) containing 2 µg/mL arachidonic acid and 10 µg/mL linoleic acid, 10 µg/mL linolenic acid, 10 µg/mL myristic acid (MA), 10 µg/mL palmitic acid (PA), and 10 µg/mL stearic acid (SA) (L0288, Sigma‐Aldrich) or 50 nM of each fatty acid, MA(130‐03432, FUJIFILM Wako Pure Chemical Industries, Ltd.), PA (165‐00102, FUJIFILM Wako Pure Chemical Industries, Ltd.), or behenic acid (BA) (216941‐5G, Sigma‐Aldrich). Then, the sperm were incubated for 30 min at 37°C under a humidified atmosphere of 5% CO_2_. Before using the fatty acids in thawing media, they were dissolved in 99.5% ethanol (09‐0770‐3, Sigma‐Aldrich) and diluted with BO media to a final concentration of 50 nM.

### 2.3 Sample preparation for gas chromatography‐mass spectrometry (GC‐MS) analysis

Fatty acids in sperm were extracted and methylated using the fatty acid methylation kit (06482‐04, Nacalai Tesque), and then purified using the Fatty Acid Methyl Ester Purification Kit (06483‐94, Nacalai Tesque), according to the manufacturer's instructions. The methylated samples (3.0 mL) were dried and dissolved in 50 μL of the elution solution. The samples were subjected to GC‐MS.

### 2.4 Gas chromatography‐mass spectrometry analysis

The measurement of fatty acids was made using Agilent 7890A GC System coupled to a JMS‐T 100 GCv mass detector at the Natural Science Center for Basic Research and Development (N‐BARD), Hiroshima University. Samples (1.0 µL aliquots) were injected in an HP‐5 capillary column (60 mm × 0.32 mm i.d. × 0.25 μm film thickness; 19091J‐413, Agilent Technologies). The measurements were performed as previously described^24^ with the following modifies: Helium was used as the carrier gas at a constant flow rate of 2.0 mL/min. The temperature of the GC oven was gradually increased from 100°C to 300°C at a rate of 10°C/min and held at 300°C for 20 min. Ionization was performed using electron ionization at an electron energy of 70 eV. Mass spectra were obtained in the scan mode (mass scanning range of 29‐800 m/z). NIST MS Search v. 2.0 was used to detect and identify the FAs.

### 2.5 Detection of Sperm Motility using a Computer‐Assisted Sperm Analysis (CASA) System

Sperm motility was evaluated using CASA as described in our previous study.^10^ A volume of 10 µL of the sample was placed in a pre‐warmed counting chamber for the CASA reading after incubation of sperm at different time intervals. Sperm tracks (0.5 s, 45 frames) were captured at 60 Hz according to our previous study^10^ using a CASA system (HT CASA‐CerosII; Hamilton Thorne). A minimum of three replicates were used for each sample, and more than 200 trajectories were recorded.

### 2.6 Mitochondrial activity

Mitochondrial activity of sperm was measured using the MitoPT® JC‐1 Assay Kit (911, Immuno Chemistry Technologies) according to Zhu *et al* 2019.^10^ Briefly, sperm samples were incubated with 200 µL of 1X working solution at 37°C for 30 min in the dark. Mitochondrial activity was analyzed using flow cytometry by using a filter with a bandwidth of 574/26 nm (Attune® NxT Acoustic Focusing Cytometer, Invitrogen) and measured as mean fluorescence intensity (MFI) of JC‐1 orange aggregates. A total of 20,000 sperm events were analyzed.

### 2.7 Oxygen consumption assay

The cellular oxygen consumption rate (OCR) was monitored in real time using a Seahorse Bioscience Extracellular Flux Analyzer (XF HS Mini, Agilent). For the flux analyzer, NaHCO_3_‐Free HTF medium was prepared according to the method used by Balbach *et al* ^25^ Frozen bovine sperm was thawed as described above. After washing with NaHCO_3_‐Free HTF medium, the cell numbers were quantified and diluted to 3,000,000 sperm cells/180 µL of NaHCO_3_‐Free HTF medium. Assay plates were coated with concanavalin A (0.5 mg/mL, FUJIFILM Wako Chemicals) overnight the day before the assay. Each well was then seeded with 180 µL of NaHCO_3_‐Free HTF medium containing 3,000,000 sperm cells. The assay was performed in 6 min cycles of mix (3 min) and measurement (3 min) steps, as per the manufacturer's recommendations. Cells were treated as indicated with myristic acid (final concentration: 5, 50, or 500 nM) and/or etomoxir (final concentration: 40 nM).

### 2.8 Flow cytometry for quantifying the intake of palmitic acid (PA)

To detect the fluorescence intensity in each sperm sample using flow cytometry, frozen‐thawed bovine sperm was incubated with fluorescently labeled palmitic acid (BODIPY^TM^ FL C_16_, D3821 Invitrogen, USA) in a dose‐dependent manner (1, 10, and 100 µM). After washing, the fluorescence level was analyzed using flow cytometry by using a filter with a bandwidth of 530/30 nm (Attune® NxT Acoustic Focusing Cytometer, Invitrogen) and measured as the intensity of the average value. A total of 20,000 sperm events were analyzed.

### 2.9 Immunofluorescence

Sperm was thawed and washed with BSA‐free BO medium, mounted on glass slides, and air‐dried. Sperm was fixed with 100% methanol for 10 min at room temperature. Then, it was probed with a 1:100 diluted primary antibodies (Anti‐CD36 antibody (NB 110‐59724, Novus Biologicals) and anti‐GOT2 antibody (NBP2‐16708, Novus Biologicals). After washing with PBS, the antigens were visualized using Cy3‐conjugated goat anti‐mouse IgG (1:100, Sigma), FITC‐conjugated goat anti‐rabbit IgG (1:100, Sigma), and DAPI (VECTESHIELD Mounting Medium with DAPI, Vector Laboratories). Digital images were captured using a Keyence BZ‐9000 microscope (Keyence Co.).

### 2.10 Statistical analysis

Statistical analyses of data from three or four replicates were performed for comparison using either Student's t test or one‐way ANOVA followed by Student's t test (Statview; Abacus Concepts, Inc).

## 3 RESULTS

### 3.1 Sperm motility parameters with the lipid mixture (LM)

**FIGURE 1:**
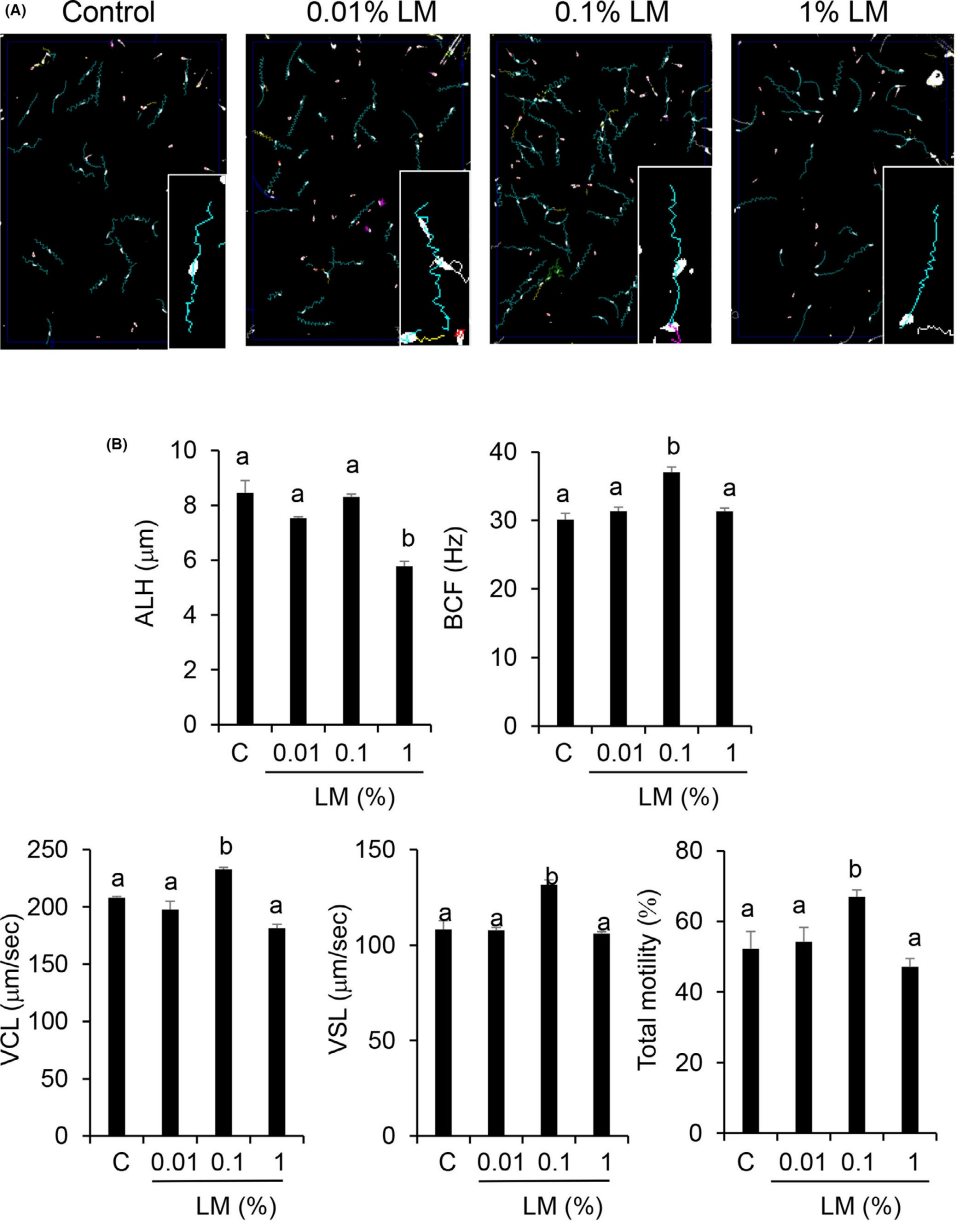
(A) CASA‐derived changes of sperm motility, which was tracked with different doses of lipid mixture (LM) and control. (B) LM improves the sperm motility pattern. Here, LM means thawing sperm with lipid mixture and control means thawing sperm without lipid mixture. Values are presented as mean ± SD of three replicates. Different superscripts are significantly different at *P* <.05

Sperm motility parameters were calculated using the CASA‐derived motility tracks after 30 min of incubation. Beat cross frequency (BCF), curvilinear velocity (VCL), straight‐line velocity (VSL), and total motility (TM) were significantly (*P* <.05) higher in sperm thawed with 0.1% (v/v) LM than in sperm thawed without the LM (control) and other doses of the LM (Figure 1). The lateral amplitude (ALH) was not significantly changed in all conditions, except for the 1% LM conditions. We selected 0.1% LM as the optimum dose for further experiments.

We tried to create scatter plots of individual sperm data considering the parameters that were significantly increased by LM treatment, BCF (y‐axis of graph), VSL (x‐axis of graph), BCF (y‐axis of graph), VCL (x‐axis of graph), VSL (y‐axis of graph), and VCL (x‐axis of graph), to understand the effect of the LM on individual sperm (Supplemental Figure 1). In each figure, we created the lines in the scatter plot considering the mean value of each motility parameter in the control group of sperm. The percentage of sperm in the upper right quadrant of the scatter plot was significantly (*P* <.05) higher in the LM treatment group than in the control group in both figures.

### 3.2 Effect of LM on sperm membrane fatty acids

**FIGURE 2:**
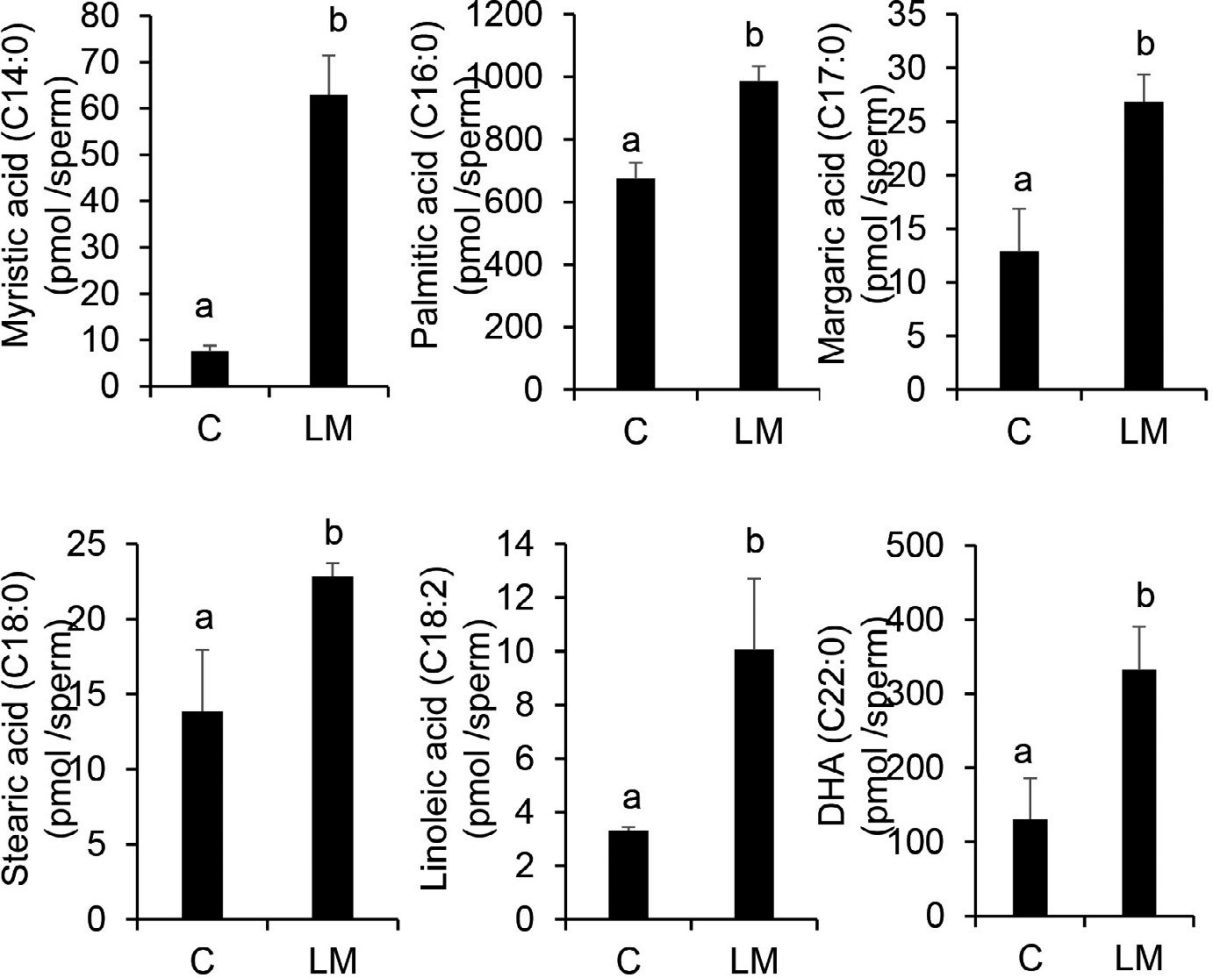
Total fatty acids (pM/ Sperm) after thawing the sperm in lipid mixture (LM) compared to the control (LM‐), obtained using GC‐MS. Y‐axis indicates the amount of total fatty acids. Values are presented as mean ± SD of three replicates. Different superscripts are significantly different at *P* <.05. DHA, docosahexaenoic acid

To understand whether sperm selectively takes up fatty acids from exogenous sources, sperm were thawed using 0.1% (v/v) of a LM containing fatty acids and 0.22 mg/mL cholesterol. Fourteen types of fatty acids were detected using GC‐MS (Supplemental Figure 2), and six of them were significantly (*P* <.05) increased in sperm thawed with LM, compared with sperm without LM (Figure 2).

### 3.3 Sperm motility with different fatty acids

**FIGURE 3:**
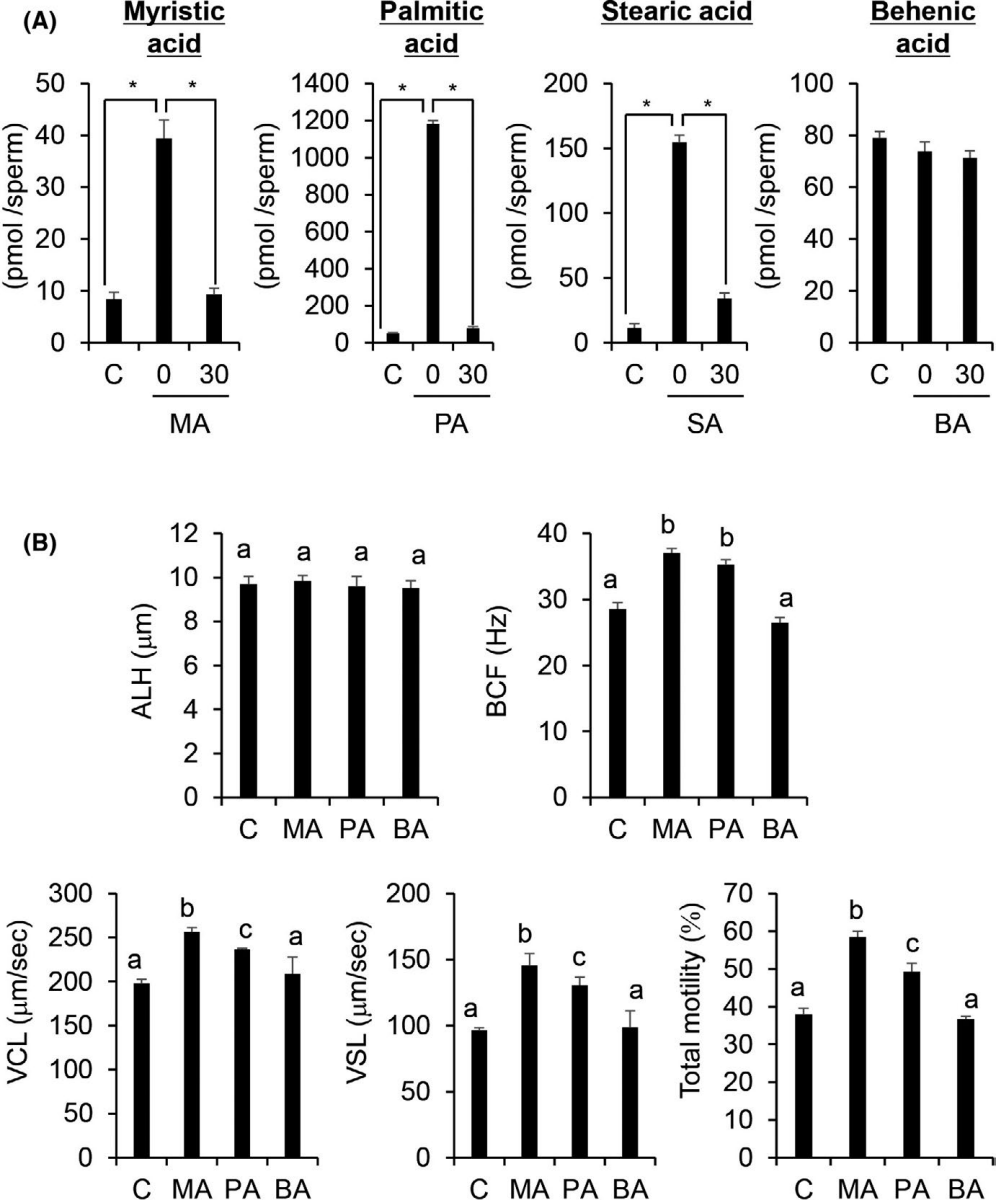
(A) Total fatty acid amount measured using GC‐MS after thawing the sperm in the respective fatty acids and after incubation for 30 minutes at 37°C. Y‐axis indicates the amount of fatty acids (pM/sperm). Values are presented as mean ± SD of three replicates. Different superscripts are significantly different at *P* <.05. Control = without any fatty acids. (B) Fatty acids improved sperm motility parameters. Values are presented as mean ± SD of three replicates. Different superscripts are significantly different at *P* <.05. MA, myristic acid (C14:0); PA, palmitic acid (C16:0); BA, behenic acid (C22:0); and C, without any fatty acids. All fatty acids were used at 50 nM

We tested whether the exogenous fatty acids of sperm were incorporated into sperm and then used as energy sources during 30 min of incubation to increase sperm velocity. GC‐MS showed a significant increase in the levels of MA, PA, and SA, but not behenic acid (BA), in sperm treated with each fatty acid. Interestingly, the increasing levels were not maintained during incubation, and the levels were significantly decreased to the basal levels. However, BA showed no significant changes during incubation (Figure 3A).

In the sperm treated with MA or PA, BCF, VCL, VSL, and TM were significantly (*P* <.05) higher than those in the control (F‐) group (Figure 3B). The values of VCL, VSL, and TM were the highest in the MA group. We considered the relationship between BCF (y‐axis of graph) and VSL (x‐axis of graph), between BCF (y‐axis of graph) and VCL (x‐axis of graph), and between VSL (y‐axis of graph) and VCL (x‐axis of graph). We created the lines in the scatter plot considering the mean value of each motility parameter in the control group of sperm. The percentage of sperm in the upper right quadrant of the scatter plot was significantly (*P* <.05) higher in the fatty acid treatment group than in the control group (Supplemental Figure 3).

### 3.4 LM and fatty acids affect sperm mitochondrial activity

**FIGURE 4:**
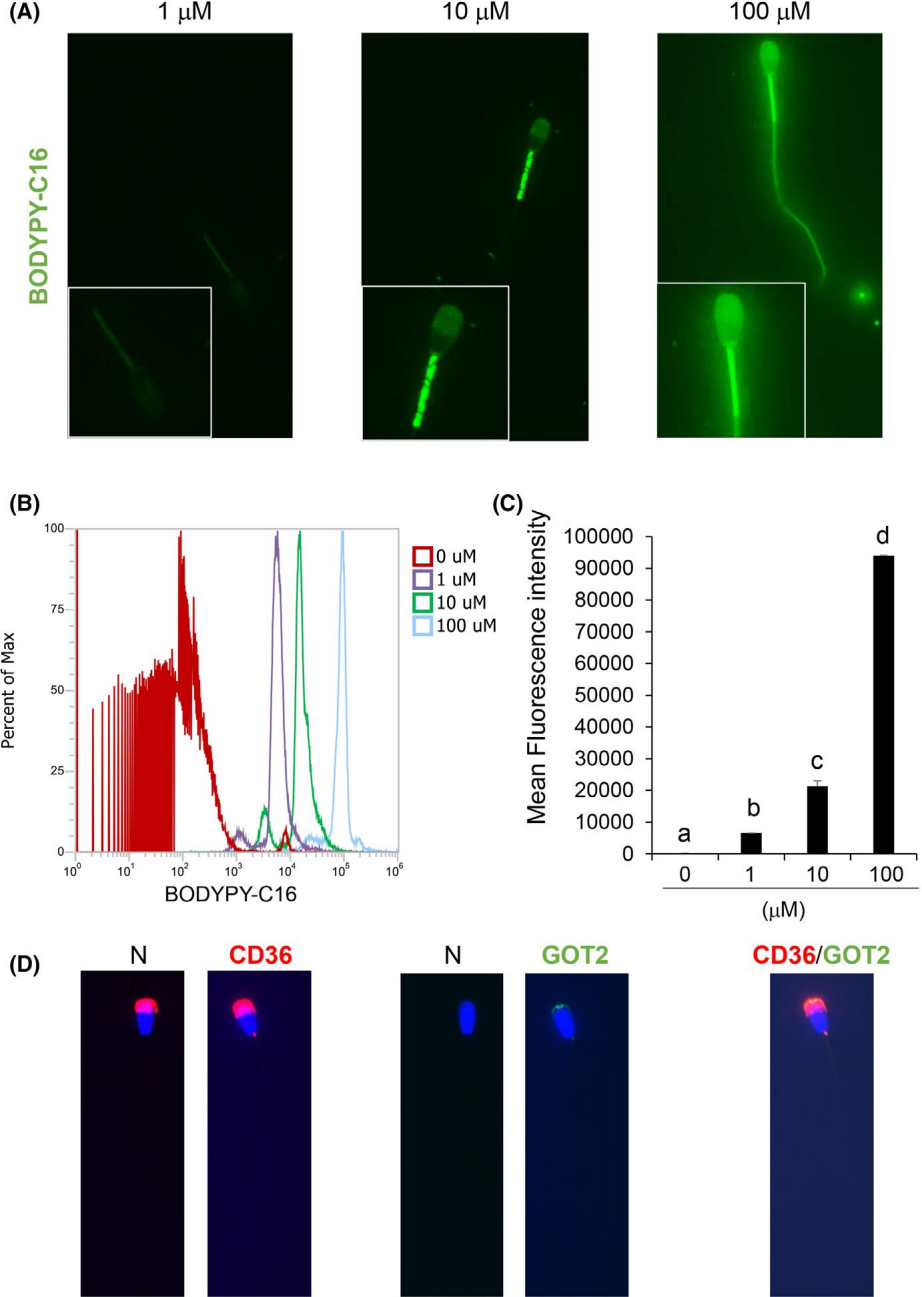
(A) Sperm fluorescence signal with BODYPY‐C16 at different concentrations. (B) Overlay of fluorescence intensity peak with BODYPY‐C16 at different concentrations. (C) Mean fluorescence intensity of sperm at different concentrations of BODYPY‐C16. (D) Localization of fatty acid transporter based on CD36 and GOT2. Negative controls (N) were performed without primary antibody. Red signal in negative control of CD36 was not specific signal against primary antibody

When frozen‐thawed sperm were incubated with fluorescently labeled palmitic acid, fluorescence signals were selectively observed in the midpiece of sperm. The fluorescence intensity in each sperm detected using FACS was significantly increased in a dose‐dependent manner (Figure 4A, B, and C). Furthermore, fatty acid transporters (CD36 and GOT2) were localized in the joint region between the head and the midpiece of sperm (Figure 4D).

**FIGURE 5:**
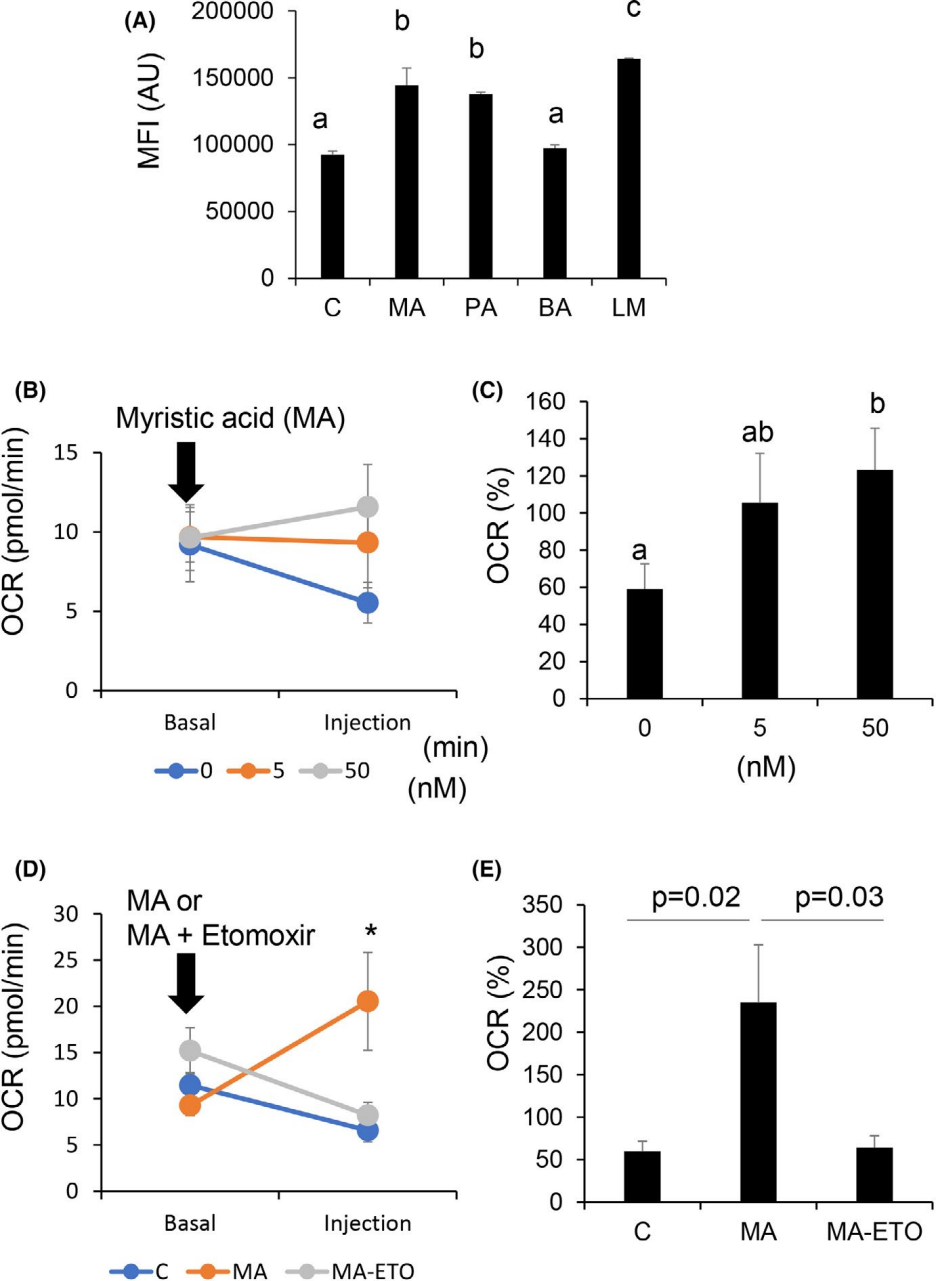
Mitochondrial activity and metabolic effect of myristic acid (MA) or Etomoxir, the inhibitor of β‐oxidation, on frozen bovine sperm. Values are presented as mean ± SD of three replicates. Different superscripts are significantly different at *P* <.05. MA, myristic acid (C14:0); PA, palmitic acid (C16:0); BA, behenic acid (C22:0); LM, lipid mixture (0.1%); and control, without lipid mixture or fatty acids. (A) Mean fluorescence intensity of mitochondrial activity with different fatty acids and lipid mixtures based on JC‐1 staining. All fatty acids were used at 50 nM. (B) Tracing of OCR before and after treatment with several concentrations of myristic acid (MA). (C) Increase in OCR after treatment with MA. (D) Tracing of OCR before and after treatment with 50 nM MA or MA + Etomoxir. (E) Increase in the ratio of OCR after treatment with 50 nM MA or MA + Etomoxir

The MFI of JC‐1 staining in sperm was significantly increased by treatment with MA and PA, but not with BA (Figure 5A). Significant induction was also observed after treatment with LM. To quantify the metabolic changes in mitochondrial activity, we monitored real‐time changes in mitochondrial β‐oxidation using a Seahorse Bioscience Extracellular Flux Analyzer. First, we traced the OCR at different concentrations of MA (Figure 5B and C). A significant increase in OCR was induced by the injection of 50 nM MA. Etomoxir, an inhibitor of β‐oxidation, significantly suppressed the MA‐induced OCR rate (Figure 5D and E).

## 4 DISCUSSION

Sperm motility patterns change from ejaculation to the fertilization process. In in vivo fertilization, the motility pattern of sperm is altered from a linear motility in the uterus to a zigzag motility in the oviduct during the journey of sperm in the female reproductive tract.^2^ It is generally accepted that sperm motility is regulated by ATP, which is produced either from the glycolytic pathway in the cytoplasm, OXPHOS in mitochondria, or from both.^10^, ^26^ In a boar sperm study, mitochondrial activity was increased under low‐glucose conditions, and the ATP produced in mitochondria was associated with a high‐speed linear motility,^10^ which is characterized by high straight‐line velocity (VSL) and a low lateral amplitude (ALH),^4^ indicating that sperm linear motility is directly associated with mitochondrial activity.

In our lipid mixture (LM) experiments, progressive linear motility was improved in frozen bovine sperm with high straight‐line velocity (VSL). A high curvilinear velocity (VCL) was also observed in our LM treatment compared to the control; however, ALH was not increased, suggesting that hyperactivation was not induced by the treatment with the LM because both ALH and VCL are increased, whereas VSL is decreased in hyperactivated sperm.^10^ Individual sperm effects of the LM on sperm motility parameters also revealed that the LM significantly affected (*P* <.05) individual sperm VSL and VCL, indicating that a linear motility pattern was induced after the treatment with the LM.

As lipid mixtures are composed of both saturated and unsaturated fatty acids and cholesterol, our present study showed that the levels of saturated long fatty acids, including myristic acid (C14), palmitic acid (C16), margaric acid (C17), and stearic acid (C18), were increased in sperm after treatment with the LM. It has been reported that unsaturated fatty acids reduce the quality of frozen‐thawed bull sperm in a citrate extender.^27^ Similarly, other experiments revealed that unsaturated fatty acid supplementation lowered sperm viability, motility, and morphology.^28^ Therefore, we added saturated fatty acids to the sperm thawing medium to determine whether exogenous saturated fatty acids would be incorporated into frozen‐thawed bull sperm and improve the motility of frozen‐thawed sperm. The addition of long‐chain saturated fatty acid, myristic acid (C14), but not ultralong fatty acid, behenic acid (C22), to the thawing medium significantly (*P* <.05) induced a linear motility pattern. It has also been reported that long‐chain saturated fatty acids, palmitic acid (C16), can preserve progressive linear motility and viability while maintaining the ROS levels at a minimum level in boar sperm.^20^ In contrast, the very long‐chain saturated fatty acids (C22:0 and C24:0) were inversely related to the total antioxidant capacity, which is directly correlated with motion parameters after thawing human sperm.^28^ Our saturated fatty acid treatment in thawing media of frozen bovine sperm improved the progressive linear motility, suggesting that saturated fatty acid could be utilized in the mitochondria of sperm for the production of ATP.

Although fatty acids are essential nutrients for cellular functions,^29^ their transport across the plasma membrane in sperm is not well understood. There is evidence that the uptake of protein‐facilitated fatty acids is the key pathway in metabolic tissues, including the liver, adipose tissue, and muscle.^30^, ^31^ CD36 is a major participant in metabolic tissues, including proteins involved in fatty acid uptake.^32^, ^33^ CD36 forms a heterodimer with plasma membrane fatty acid‐binding protein (FABPpm/GOT2), which transports vital fatty acids in the heart of humans.^34^ Another study on rat liver cells revealed that FABPpm/GOT2 is located in the plasma membrane and has been detected as a fatty acid transporter that regulates the uptake of long‐chain fatty acids.^35^ Our immunofluorescence results showed that both CD36 and GOT2 are localized in the joint of the head and midpiece of sperm, where mitochondria are actually located. Moreover, fluorescence‐tagged palmitic acid was observed in the sperm midpiece, indicating that saturated fatty acids were taken up to the sperm midpiece in a CD36‐GOT2‐dependent manner for ATP production in mitochondria.

It has been reported that exogenous fatty acids can be used as energy substrates for sperm. For example, a boar sperm study reported that the addition of exogenous fatty acids (oleic acid and palmitic acid) to the dilution medium improved sperm quality, since these were used as energy substrates for ATP production via β‐oxidation.^20^ In our study, myristic acid showed good results in mitochondrial β‐oxidation, suggesting that long‐chain saturated fatty acids in thawing media of frozen bovine sperm can be used as an energy substrate for ATP production in mitochondria in the sperm midpiece to induce and maintain their linear motility.

The sperm motility was improved by the treatment with lipid mixture or each type of saturated fatty acid in this study. The positive effects of lipid mixture were detected in a dose‐dependent manner; however, the maximum effects were observed at 0.1% and a high concentration of lipid mixture (1%) significantly decreased sperm motility parameter. When mitochondria ATP production is activated, ROS generation is also induced as by‐products in not only sperm but also other cells.^36^ The oxidative stress damages mitochondria proteins that are encoded by nuclear genome in boar sperm.^37^ Because transcription and translation are not activated in sperm nucleus, it is hypothesized that the damaged proteins would be not turned over. Thus, to maintain the high sperm motility induced by fatty acids, the exogenous antioxidant would be required for keeping mitochondria proteins in the hyper‐activated mitochondria.

**FIGURE 6:**
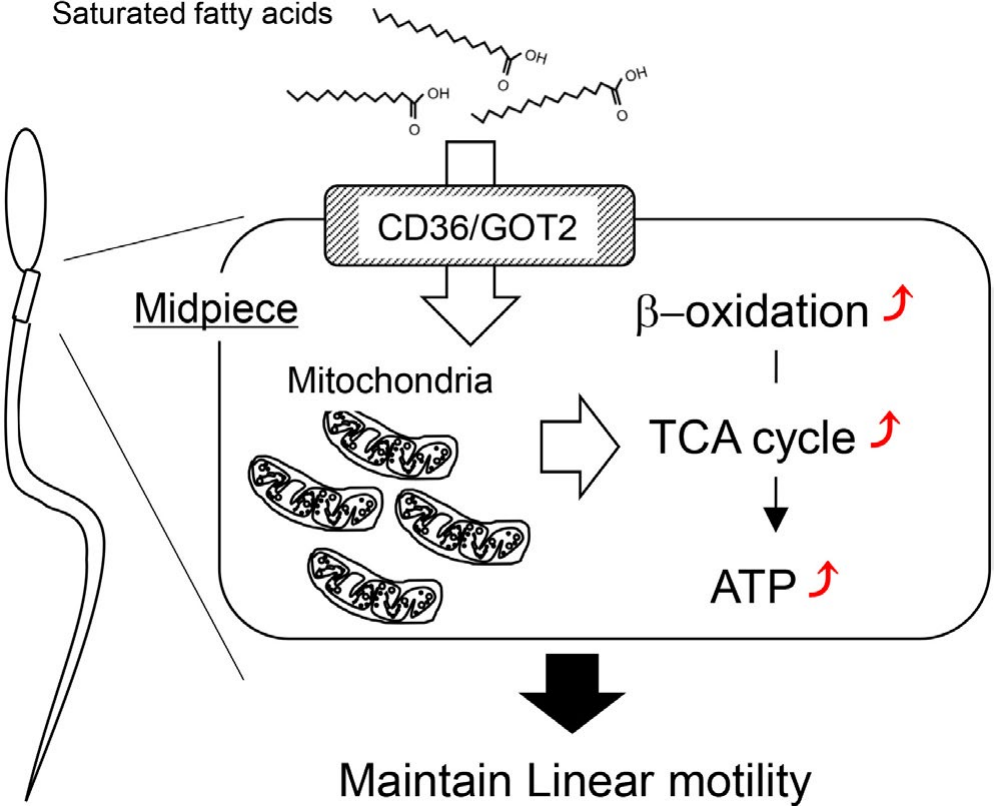
Systematic image of the effect of saturated fatty acids. Saturated fatty acids are transported to the sperm midpiece through fatty acid transporters (CD36/GOT2), and then are used as energy substrates for mitochondrial β‐oxidation to activate tricarboxylic acid cycle (TCA) cycle. By this system, ATP is produced, which impacts on the linear motility of frozen bovine sperm

In conclusion (Figure 6), it is clear that saturated fatty acids are transported to the sperm mitochondria through fatty acid transporters, where they act as energy substrates for mitochondrial β‐oxidation to produce energy for the sperm to maintain linear motility. Thus, to increase and maintain the sperm fertilization ability, exogenous saturated fatty acids are beneficial for inducing and maintaining sperm linear motility. It may be possible that thawing sperm with saturated fatty acids containing insemination medium will boost sperm linear motility in the female genital tract to improve fertilization, as a simple and low‐cost tactic.

4.1

## CONFLICT OF INTEREST

Masayuki Shimada received salary from Saint Mother Obstetrics and Gynecology Clinic as an adviser, and from Hiroshima Cryopreservation Service CO as a director. Md. Mazharul Islam, Takashi Umehara, and Natsumi Tsujita declare no conflict of interests.

Human and animal rights: This article does not contain any studies with human subjects and animal subjects performed by the any of the authors.

## Supporting information

Fig S1‐S3
