# Supplementary material for: Saturated fatty acids accelerate linear motility through mitochondrial ATP production in bull sperm
Source: Reprod Med Biol. 2021 May 6;20(3):289–98. doi: 10.1002/rmb2.12381 (PMC8254171; doi:10.1002/rmb2.12381)
Supplement: Supplementary file 1 — Fig S1‐S3 [file RMB2-20-289-s001.pdf]

(A)

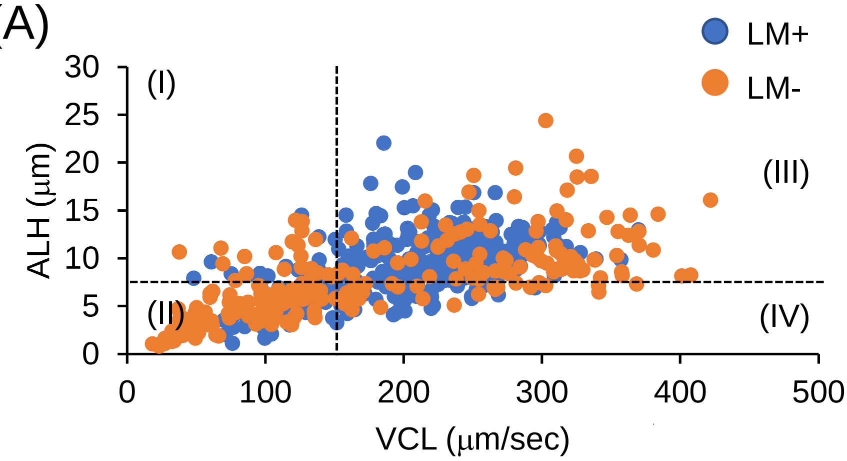

	Sperm (%) (Mean +- SEM)	
	LM-	LM+
I	13.3+-1.5	13.0+-4.0
II	40.0+-1.7	14.0+-1.5 *
III	35.3+-1.3	60.0+-5.0 *
IV	10.6+-1.9	13.3+-2.3

(B)

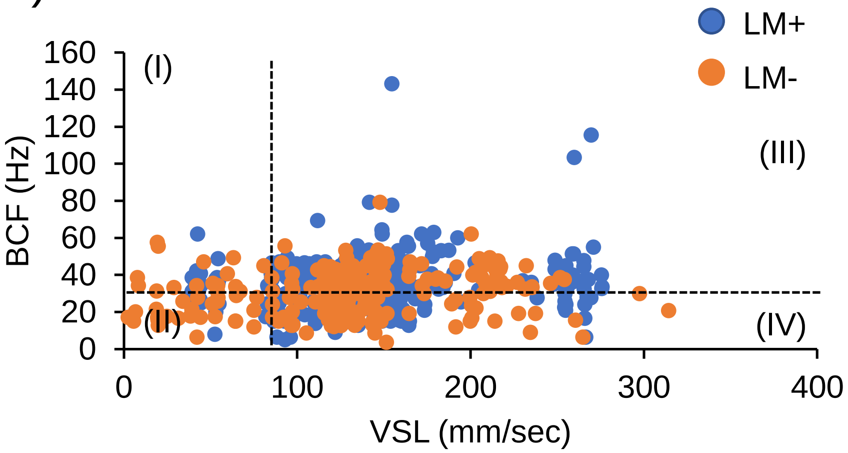

	Sperm (%) (Mean +- SEM)	
	LM-	LM+
I	20.7+-2.3	18.0+-3.2
II	33.3+-3.3	14.0+-1.2 *
III	26.7+-2.1	51.0+-6.7 *
IV	14.3+-1.8	17.0+-3.8

(C)

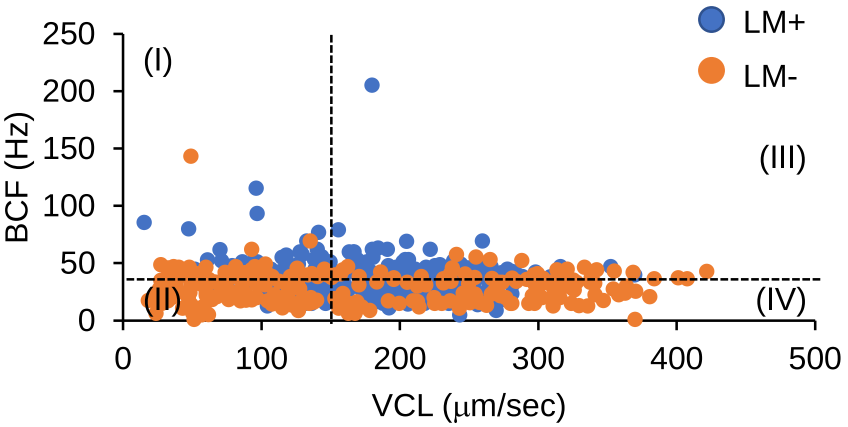

	Sperm (%) (Mean +- SEM)	
	LM-	LM+
I	31.0+-2.1	22.0+-1.8
II	25.3+-1.2	10.7+-1.8 *
III	20.7+-1.5	44.7+-1.8 *
IV	20.7+-2.3	22.7+-2.4

(D)

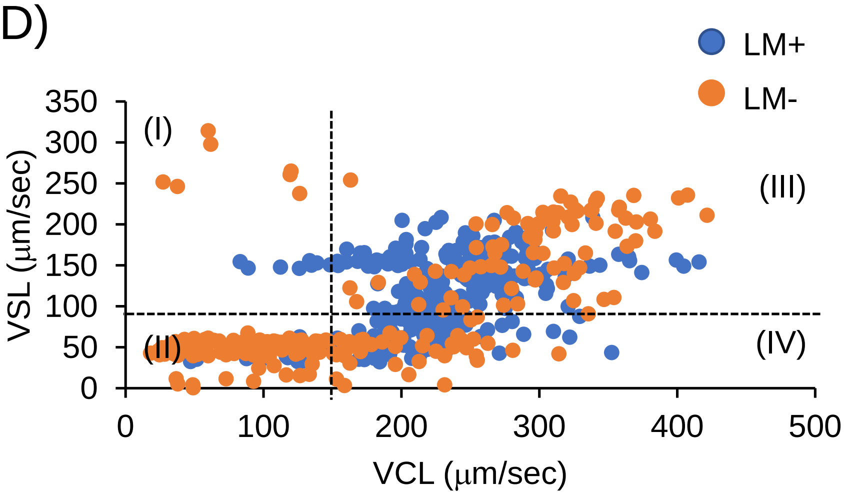

	Sperm (%) (Mean +- SEM)	
	LM-	LM+
I	5.0+-1.2	5.3+-0.9
II	51.7+-2.4	6.3+-0.9 *
III	26.7+-1.5	61.3+-1.8 *
IV	12.0+-1.7	26.0+-1.2

Supplemental Figure 1

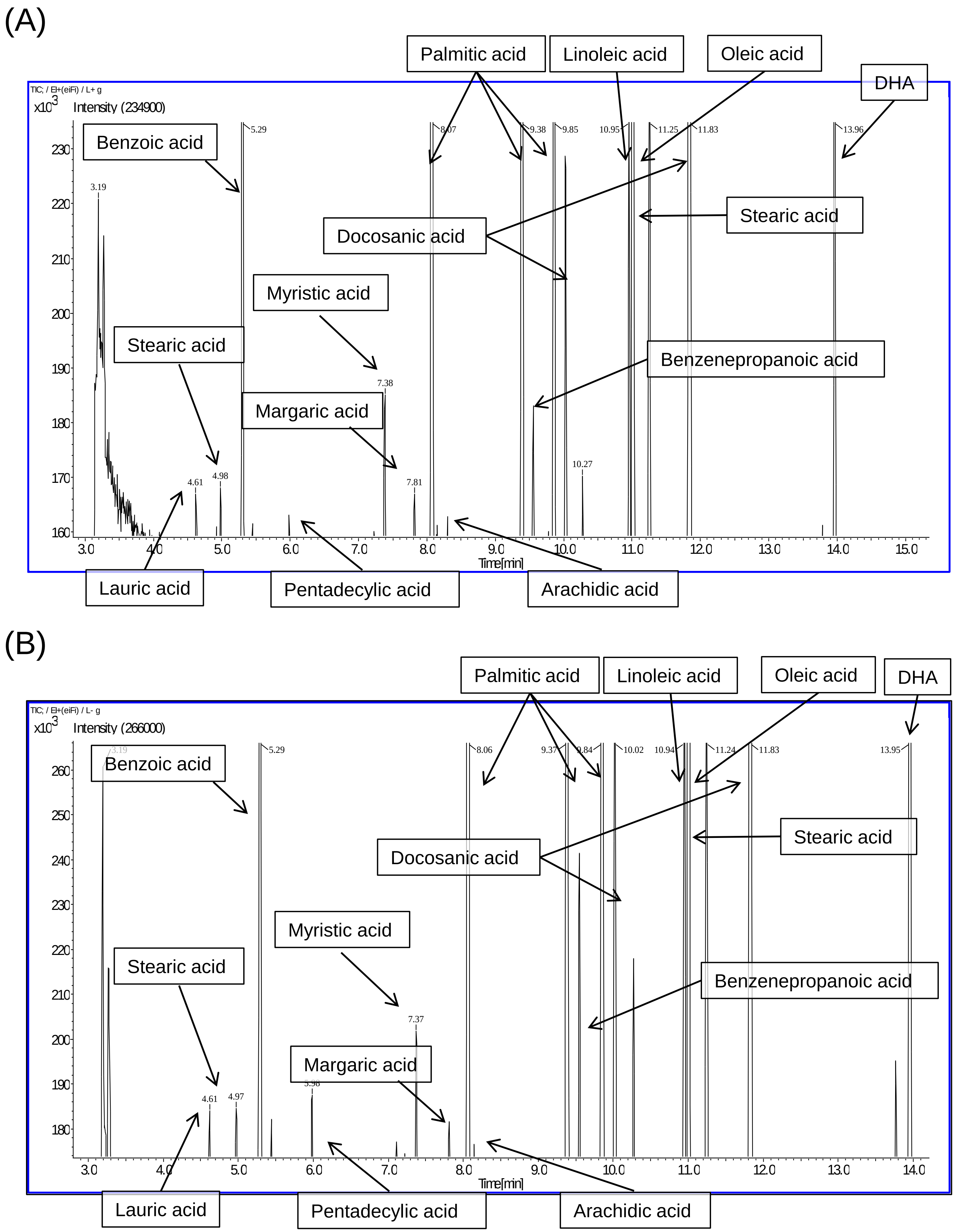

Supplemental Figure 2

(A)

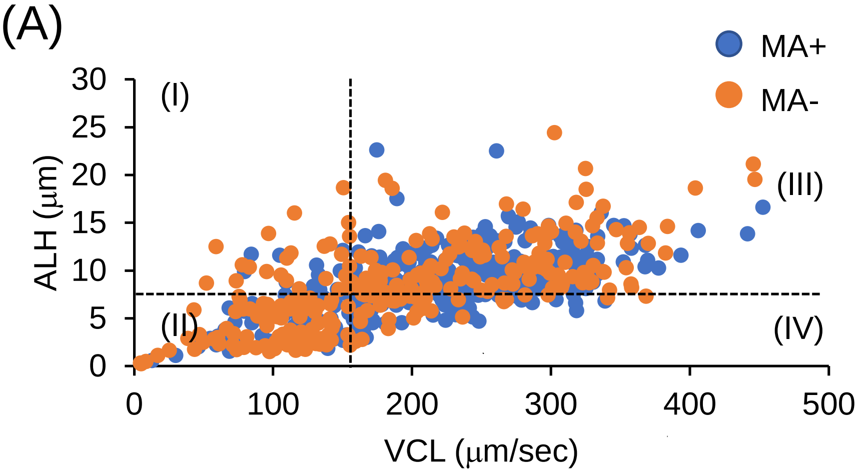

	Sperm (%) (Mean +- SEM)		
	MA-	MA+	
I	12.8+-1.2	12.7+-2.8	
II	42.7+-2.7	22.3+-2.8	*
III	32.7+-2.8	51.7+-2.4	*
IV	13.0+-2.0	14.0+-1.7	

(B)

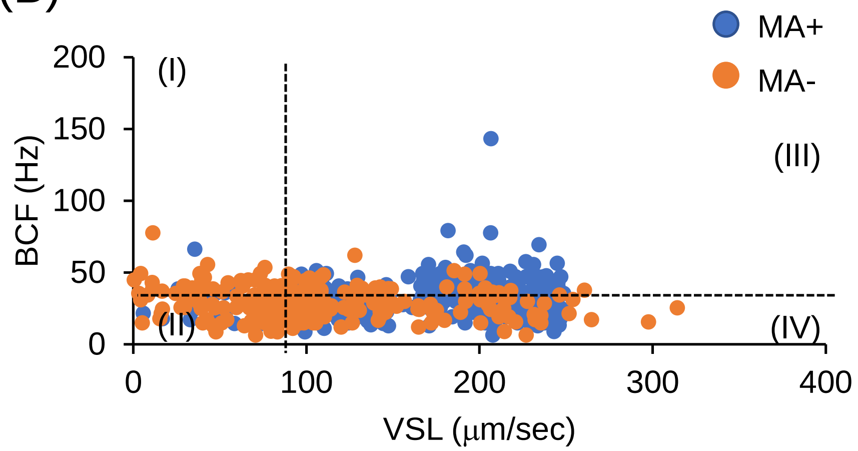

	Sperm (%) (Mean +- SEM)		
	MA-	MA+	
I	27.0+-5.0	9.0+-1.5	
II	35.0+-4.7	13.7+-1.8	*
III	23.0+-4.6	55.3+-6.6	*
IV	15.3+-3.7	21.3+-5.7	

(C)

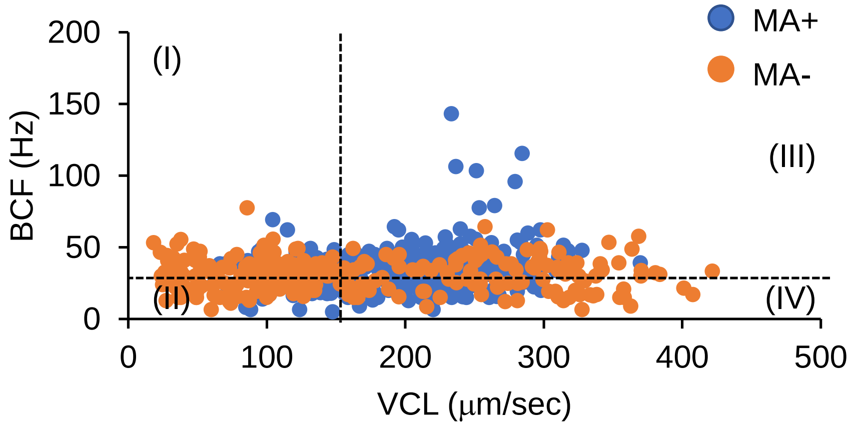

	Sperm (%) (Mean +- SEM)		
	MA-	MA+	
I	30.3+-1.5	11.0+-1.5	
II	30.0+-1.7	12.0+-1.2	*
III	21.7+-0.9	51.7+-2.0	*
IV	19.0+-1.2	25.7+-1.8	

(D)

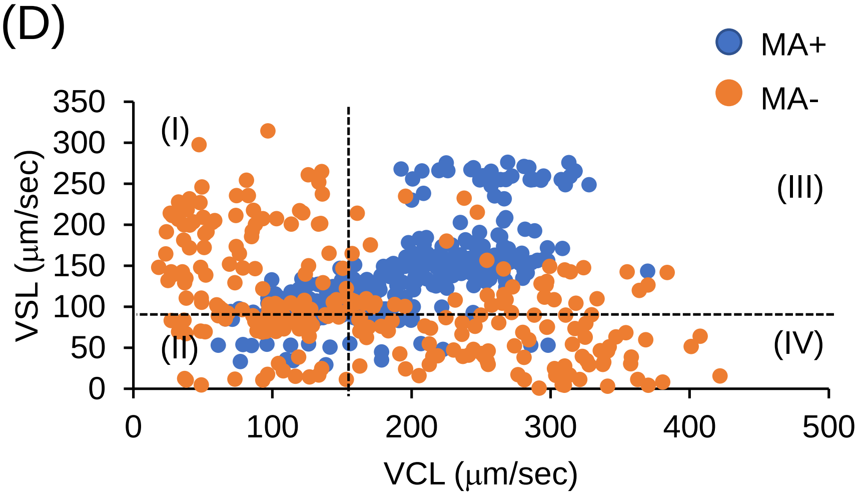

	Sperm (%) (Mean +- SEM)		
	MA-	MA+	
I	30.0+-1.0	10.0+-1.2	
II	30.0+-1.2	11.7+-0.9	*
III	10.3+-0.9	67.3+-1.2	*
IV	30.0+-1.7	8.7+-1.2	

Supplemental Figure 3
